# Causes of Dental Caries and Prophylaxis

**Published:** 1898-11

**Authors:** J. Milton Rosenthal


ARTICLE III.

### CAUSES OF DENTAL CARIES AND PROPHYLAXIS.

J. MILTON ROSENTHAL, D. D. S.

Read before the Isaac Knapp Dental Coterie, Fort Wayne, Ind., April 19, 1898.

This subject, as you know, is widely treated, and it is from past research that I desire to gather such a rude outline of the work as you have permitted me to present to you to-night.

Of all the pathological conditions of the human body, dental caries is the most frequent, 999 cases out of every 1,000 needing the attention of a dentist.

There are always two factors to be considered in disease of the teeth, as well as other structures of the body, the predisposing and the exciting causes; I shall therefore begin with the predisposing causes of dental caries.

In hereditary causes, the most important factor we find is transmission of form and shape, and the structural properties of the teeth from generation to generation, resulting in peculiarities of shape and irregularities in shape and position.

Congenital causes arise from lack of nutrition before birth, parents partaking of improper food, etc. There has been a great deal said and written for and against gestation as a cause of dental caries; certain it is that this condition does have an effect upon the teeth, and there are some grounds for the popular saying that every baby costs a tooth. This effect upon the teeth is probably brought about by diminished alkalinity of the blood and occasional tendency to acid in the saliva. Frequent vomiting with acid secretions from the stomach may also be considered a factor. Digestive disturbances at a time when demands are made upon the system for lime salts, as well as other principals of nutrition, may also be taken into consideration under this head. Many cases of early decay of the teeth due to injudicious artificial feeling might be cited.

Deleterious effects are produced upon the teeth in diseases of children, such as are caused by defective nutrition or by improper feeding and consequent impairment of the digestive functions, where food may be digested but not properly assimilated, causing lack of lime salts, and in eruptive fevers, skin diseases, gastric troubles, vitiated conditions of the saliva and in diseases of the mucous membrane.

Drugs, particularly those injudiciously or ignorantly prescribed by physicians, leave their irresistible effects. How often we hear of such a remedy; vinegar well seasoned with salt and pepper as a gargle for sore throat. Many drugs might be mentioned, such as mercurial salts, all the acids and preparations of iron, that are deleterious to tooth structure.

That there is little or no relation between density of the teeth and progress of caries has been demonstrated by Prof. Black and others, and the sooner the profession awakens to a realization of this fact the better it will be for our patients. Thousands of useful teeth have been lost because of this fallacy; and, until the profession recognizes that caries of the teeth is a disease influenced by external conditions, rather than by inherent structure of the teeth, thousands more will be consigned to the forceps.

As the science of dentistry has progressed, many theories, such as the chemical, electrical and vital, have been advanced as to the cause of caries of the teeth. The chemical theory is that caries is produced by a mere chemical action of different agents on the tooth structure. The electrical theory is still held by many, but I do not believe it is a factor in caries. The vital theory is that caries is affected by the life principal; but a vital principle is a building up process, and supports life; therefore it is not the occasion of death or decay.

It is now almost universally held that, aside from imperfection in structure and development, micro-organisms are the causes of caries of the teeth. They owe their rapid development to the secretions, deposits, etc., of the oral cavity, and not until the tissue of the tooth had undergone a certain change—first, decalcification; second, peptonization—can they adapt it to their nourishment. The decalcification is produced chiefly by acids resulting from action of the organism upon certain carbo-hydrates in the human mouth, while the peptonization is produced either by direct action of the protoplasm of the organism upon the decalcified dentine or by the action of a ferment which they produce.

Miller has found by his experiments that by infecting sterilized culture-media of various kinds with neutral saliva or with neutral carious dentine, when the culture-media contains sugar, acid is produced. By successive cultures he has isolated the organisms which produced the acid, and has found that we have two definite forms of fungi, the one always, the other probably present. The first fungus we now know produces lactic acid, and the second fungus undoubtedly does so, though the acid in the latter case is always produced in the presence of the former. These micro-organisms require sugar of some form to work upon, the fermentation is most active at 35° C to 40°C, with no production over 40°C or under 15°C. In addition to these we find many other forms, and there are probably some yet to be discovered.

The question arises, How shall we prevent these effects, and how shall we ward off the evil, that disease, from causes innumerable, shall be powerless to produce its effect and that our patients shall be possessed of teeth, sound in quality, perfect in form, unblemished even by the introduction of foreign material? Perhaps no one subject has more direct bearing on this question than has the preservation of the temporary teeth. Parents have neglected it, arguing, no doubt, that they were soon to be replaced by the permanent ones. We know that the mouth cannot be rendered aseptic and that all kinds of germs and fungi are always present. It becomes our duty therefore, in seeing that these destructive elements remain inert, to keep the teeth in such a condition that their natural protection remain proof against them.

Germs, in and of themselves—bacilli, cocci, fungi—are inoffensive, and do not harm by their mere presence, but if given a point when they may remain for a time, with the necessary environment—heat, moisture and food—they multiply at this place with great rapidity, and, in their growth, evolve or secrete substances which have the ability to dissolve chemically the constituent elements of the teeth, form gases and bring about that condition known as putrefactive fermentation. Care should be taken of the general health of children; this has a great influence upon the teeth, as well as upon the other organs of the body. They should be taught to properly masticate their food, and such food should be provided as will give sufficient exercise to the growing teeth and jaw. What foods should be recommended? It is now well known that the carbo-hydrates are the acid-forming foods, and therefore the proteids have little or nothing to do with dental caries. It will also be noticed that raw vegetable foods are less fermentable than cooked, and sugar less than starchy foods. We find the Indians, Esquimaux, and carnivorous animals that subsist on pretoid food, invariably have good teeth. Of the carbo hydrate foods, wheat flour is the most injurious, especially in the form of white bread and cakes, for which children crave. The use of oatmeal and other cereals, which have not suffered from artificial removal of their most valuable constituents, have been suggested as proper food for children. Candy, which is manufactured chiefly of glucose, a grape sugar, and saccharose, a cane-sugar, both of which are absorbed by the mucous membrane of the mouth, also finding lodgment in deep fissures and pits of the teeth, will be converted into lactic acid. While eating candy may endanger the integrity of the teeth, the consequent fermentation of starchy food is far more injurious. Caries of candy makers' teeth is probably due to the acids which are used in the manufacture of the candy, as well as to the candy eaten. The teeth of those who habitually use tobacco are, as a rule, well preserved, probably from the antiseptic property of the tobacco itself.

Nitrate of silver, by its antagonizing action to germs that produce caries, will arrest decay, being especially useful for superficial caries at the gingival margin. There are many other antiseptics that are used for this purpose, giving very satisfactory results. It is important, however, to remember that while antiseptics do destroy germs, many of them, by their irritating and solvent properties, also destroy the natural protection of the teeth against these germs, and these must be avoided.

I do not wish to take time to discuss filling materials as a means of arresting further caries of the teeth, but my opinion is that, to prevent tooth destruction where caries has begun, and to get the best possible results, gold fillings, perfectly adapted with a non-conducting lining, to prevent thermal changes, and crowns, should be used. In preserving the teeth from caries we must not lose sight of the fact that external, and not internal, agencies are the patent factors. Therefore, fillings, crowns, and other remedial attempts, must leave no rough surfaces or spaces or clefts where the secretions and germs may collect to evolve their destructive ptomaines. This embodies the whole underlying principle. The surface of the teeth must be smooth. Smoothness is their main safeguard, germs which do not find a point of attack where they may nestle undisturbed are harmless to do damage to the teeth Every mechanical or chemical action which might injure the surface should be guarded against. The crushing of enamel by the pressure of contiguous teeth when crowded, biting thread, cracking hard nuts, etc., etc., come under this head.

Roughened edges of teeth should be carefully ground and polished. I find the ground edge of a tooth polished with so-called Enamel Restorer, to do nearly, if not quite, as well as when covered with its natural smooth covering. It is more than folly to attempt to render the mouth and teeth aseptic, or even comparatively germ-free.

We should advise our patients to use such dentifirices as cleanse the teeth without mechanically injuring their surfaces; a soft brush and floss silk for removing particles which may harbor and give nutriment to germs between and on the teeth. It is better to avoid germicides altogether than to advise such preparations as have the ability even in a slight degree to chemically attack teeth and rob them of their natural protection against the causes of decay, which are always and unavoidably present in the mouth.—*Indiana Dental Journal.*

